# SCORTEN and Novel Prognostic Markers in Stevens–Johnson Syndrome/Toxic Epidermal Necrolysis: A Systematic Review and Meta‐Analysis

**DOI:** 10.1111/ajd.70082

**Published:** 2026-03-06

**Authors:** Zhao Feng Liu, Howard Yang, Lawrence Lin, Ash Tyagi, Amy Sylivris, Adithya Shastry, Nidhin Sunil Kuruvilla, Douglas Gin, Christopher Y. Chew

**Affiliations:** ^1^ Department of Dermatology The Alfred Hospital Melbourne Australia; ^2^ Department of Dermatology Monash Health Melbourne Australia; ^3^ Faculty of Medicine, Nursing and Health Sciences Monash University Melbourne Australia; ^4^ The Royal Melbourne Hospital Melbourne Australia; ^5^ Skin Health Institute Melbourne Australia

**Keywords:** drug eruptions, mortality, SCORTEN, Stevens–Johnson syndrome, toxic epidermal necrolysis

## Abstract

Stevens–Johnson syndrome/toxic epidermal necrolysis (SJS/TEN) is a rare, severe skin reaction with high mortality, most often triggered by medications. Early identification of patients at risk is essential for guiding treatment. The SCORTEN score, comprising seven clinical and laboratory variables, is widely used to predict mortality but may have limitations across different populations. We conducted a systematic review and meta‐analysis to assess SCORTEN's predictive accuracy and identify additional blood biomarkers associated with mortality in SJS/TEN. The primary aim was to evaluate the prognostic performance of individual SCORTEN parameters in adult SJS/TEN patients. The secondary aim was to explore modified SCORTEN cut‐offs and identify additional laboratory biomarkers associated with mortality. We searched Medline, Embase and the Cochrane Library up to March 2024 for relevant studies. Eligible studies were assessed for quality and pooled analyses were conducted using a random‐effects model. A total of 45 studies with 3467 patients were included. All seven SCORTEN variables were significantly associated with in‐hospital mortality. Additionally, elevated serum creatinine, a marker of kidney function, was independently associated with increased risk of death. Other biomarkers showed potential, but data were insufficient for pooled analysis. Our results reinforce the value of SCORTEN and suggest serum creatinine may enhance risk prediction. Improved prognostication in SJS/TEN could support earlier intervention and better use of critical care resources. Further research is needed to validate new biomarkers.

**Trial Registration:** PROSPERO registration number: CRD42022344691

## 1 Introduction

Stevens–Johnson Syndrome (SJS) and Toxic Epidermal Necrolysis (TEN) are a spectrum of rare and severe mucocutaneous adverse reactions, most commonly triggered by medications [1, 2, 3]. The SJS/TEN spectrum is classified by the extent of maximal epidermal detachment [4]. In SJS, skin detachment is < 10% of total body surface area (TBSA) and carries a mortality rate of approximately 10% [5]. In TEN, skin detachment is > 30% of TBSA and carries a mortality rate as high as 50% [5]. SJS/TEN overlap syndrome describes all remaining cases with skin detachment between 10% and 30% of TBSA [2].

Given the high mortality rate of SJS/TEN, significant effort has been directed towards the development of accurate prognostication tools [6, 7, 8]. The SCORTEN tool is a severity of illness score developed in 2000 by Bastuji‐Garin et al. [6] to predict the inpatient mortality of SJS/TEN. It is made up of seven clinical and laboratory variables including age ≥ 40, presence of solid organ or haematological malignancy, epidermal detachment > 10% TBSA, heart rate ≥ 120, blood urea nitrogen (BUN) > 10 mmol/L, serum bicarbonate < 20 mmol/L and serum glucose > 15 mmol/L.

Since its inception, SCORTEN has been widely applied in the clinical setting to provide prognostic information [9, 10], as well as in research studies, acting as an internal control to assess the efficacy of therapeutic interventions [11, 12]. However, recent studies have called into question the accuracy of SCORTEN in the contemporary context of specialised burn centres and immunomodulatory therapy [11, 13, 14, 15]. Attempts have been made to develop improved prognostication tools by identifying new clinical predictors of mortality and updating the cut‐off points for existing ones [7, 16].

To this end, numerous studies have attempted to evaluate the prognostic values of existing and emerging clinical and laboratory parameters [17, 18, 19, 20, 21, 22]. However, no previous meta‐analysis has evaluated the discriminatory power of individual parameters that comprise the SCORTEN score. It is necessary to assess the component strengths and weaknesses of SCORTEN to modernise its role in clinical practice and research. The primary aim of our systematic review and meta‐analysis is to examine the prognostic performance of individual SCORTEN parameters in adult SJS/TEN patients. The secondary aim is to explore the prognostic value of modified SCORTEN parameters and to identify novel prognostic markers that may provide additional information.

## 2 Methods

### 2.1 Search Strategy

The systematic review was conducted in accordance with the PRISMA reporting checklist [23]. Secondary exploratory analyses of modified SCORTEN cut‐offs and additional biomarkers were conducted to reflect developments in the literature and are reported in accordance with PRISMA guidelines. OVID Medline, Embase and Cochrane Library were systematically searched from inception until March 31st, 2024, for all SJS/TEN studies that reported at least one of the seven SCORTEN parameters. Additionally, based on a comprehensive literature review, ‘albumin’, ‘creatinine’, ‘white cell count’, ‘haemoglobin’, ‘platelet’, ‘eosinophil’, ‘bilirubin’, ‘c‐reactive protein’ and ‘lactate’ were also included in the search due to their recognised relevance in predicting outcomes in acute inflammatory conditions (Appendix S1 and Figure S1). The reference list of all included studies was further screened to identify any potentially eligible records [6].

### 2.2 Study Selection

**FIGURE 1:**
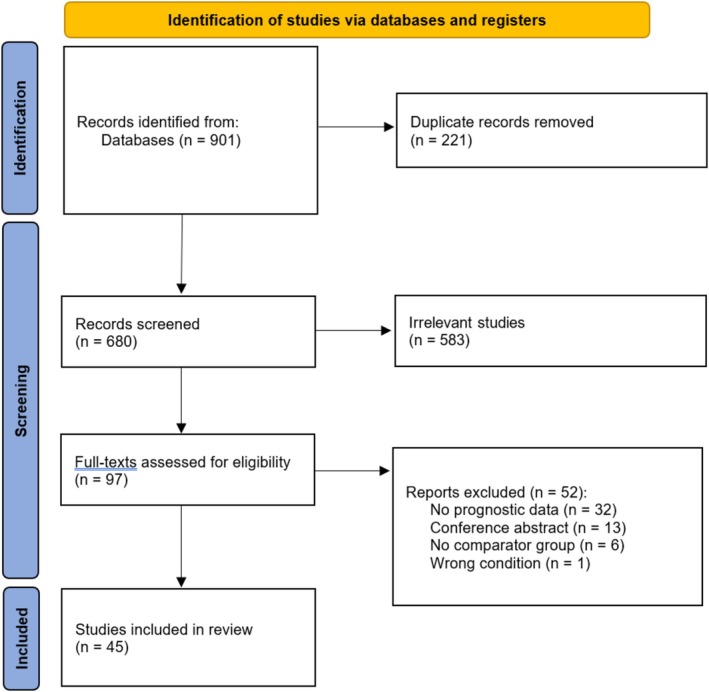
PRISMA flow diagram.

After all records were organised and duplicates excluded, title and abstract screening was conducted by two reviewers (Z.L. and A.S.) independently. Studies were included if they: (1) contain patients diagnosed with SJS/TEN, (2) contain at least one deceased and one surviving patient and (3) report extractable information about prognostic markers in the form of counts, or measures of central tendency. Following title and abstract screening, potentially eligible records were retrieved, and the full‐texts were screened by two reviewers (Z.L. and A.S.) independently (Figure 1). Disagreements regarding a study's eligibility were resolved by discussion, and if necessary, by the input of a third reviewer (C.C.).

### 2.3 Data Collection

Data were extracted by two reviewers (Z.L. and A.S.) independently using a standardised spreadsheet. Extracted data include demographic characteristics, patient outcomes and prognostic marker values. This was recorded as counts for variables with dichotomous cutoffs and in measures of central tendency (mean or median) for continuous variables. Studies with missing data on a prognostic variable were excluded from its meta‐analysis.

### 2.4 Quality Assessment

Risk of bias assessment was completed by using the Quality In Prognosis Studies (QUIPS) tool [24]. All studies were assessed across the domains of study participation in terms of recruitment and patient population, attrition of participants, predictors assessed, outcome measurement, consideration of confounding factors and statistical analysis/reporting.

### 2.5 Statistical Analysis

Statistical analysis was conducted using R statistical software (version 4.3.1; R Core Team, 2021). Demographic and clinical parameters were summarised using descriptive statistics. Meta‐analyses were performed on two fronts: one for dichotomous SCORTEN variables and another for continuous prognostic variables, both comparing deceased against surviving patients.

For continuous variables, comparisons were made using mean, standard deviation and sample size, following the methodology outlined by Borenstein et al. [25] Unstandardized effect sizes were computed using Hedge's *g* with a random‐effects model, employing the DerSimonian–Laird method and Knapp–Hartung standard error adjustment. For dichotomous SCORTEN variables, odds ratios (OR) were calculated and pooled using random‐effects models, following guidelines from the Cochrane Handbook. 95% confidence intervals (95% CI) were calculated. Statistical heterogeneity was assessed using *I* ^2^ statistics. Publication bias is represented with funnel plots (Figure S2).

## 3 Results

### 3.1 Study Characteristics

A total of 45 studies met the inclusion criteria and were included in the final analysis (Figure 1). 37 (82.2%) of these were retrospective cohort studies, with the remaining 8 (17.8%) being prospective (Table S1). Across these studies, 3467 patients were analysed, of whom 652 (18.8%) did not survive until discharge. The weighted mean age of the cohort was 48.1 years, with a slight female predilection (1:1.15). The cohort comprised 34.2% with SJS, 16.2% with SJS/TEN overlap and 24.8% with TEN.

### 3.2 SCORTEN Parameters

**FIGURE 2:**
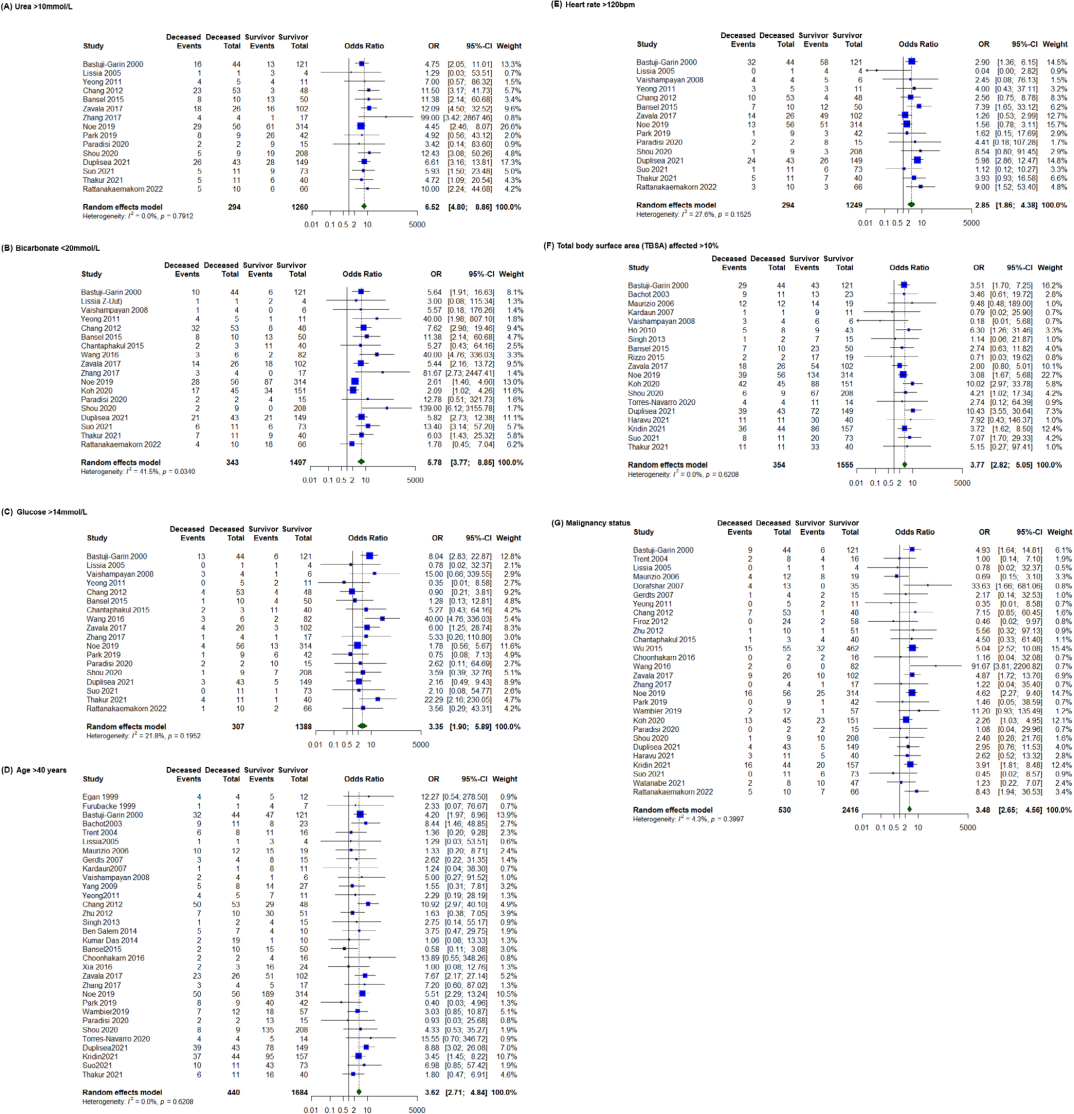
Forest plots illustrating the association between individual SCORTEN variables and mortality in Stevens–Johnson syndrome/Toxic Epidermal Necrolysis patients. (A) Urea > 10 mmol/L (B) Bicarbonate < 20 mmol/L (C) Glucose > 14 mmol/L (D) Age > 40 years (E) Heart rate > 120 bpm (F) Total body surface area (TBSA) affected > 10% (G) Malignancy status.

The seven SCORTEN parameters were analysed, including serum BUN levels above 10 mmol/L, serum bicarbonate levels below 20 mmol/L, serum glucose levels above 14 mmol/L, age exceeding 40 years, heart rate above 120 beats per minute (bpm), initial TBSA involvement exceeding 10% and history of malignancy. All seven SCORTEN parameters exhibited significant and independent associations with inpatient mortality: urea (OR = 6.52, 95% CI 4.80–8.86, *p* < 0.01, studies = 15), bicarbonate (OR = 5.78, 95% CI 3.77–8.85, *p* < 0.01, studies = 18), glucose (OR = 2.85, 95% CI 1.64–4.98, *p* < 0.01, studies = 16), age (OR = 3.62, 95% CI 2.71–4.84, *p* < 0.01, studies = 32), heart rate (OR = 2.85, 95% CI 1.86–4.38, *p* < 0.01, studies = 15), TBSA (OR = 3.77, 95% CI 2.82–5.05, *p* < 0.01, studies = 19) and malignancy status (OR = 3.48, 95% CI 2.65–4.56, *p* < 0.01, studies = 28) (Figure 2).

### 3.3 Variations to SCORTEN Cut‐Off

**FIGURE 3:**
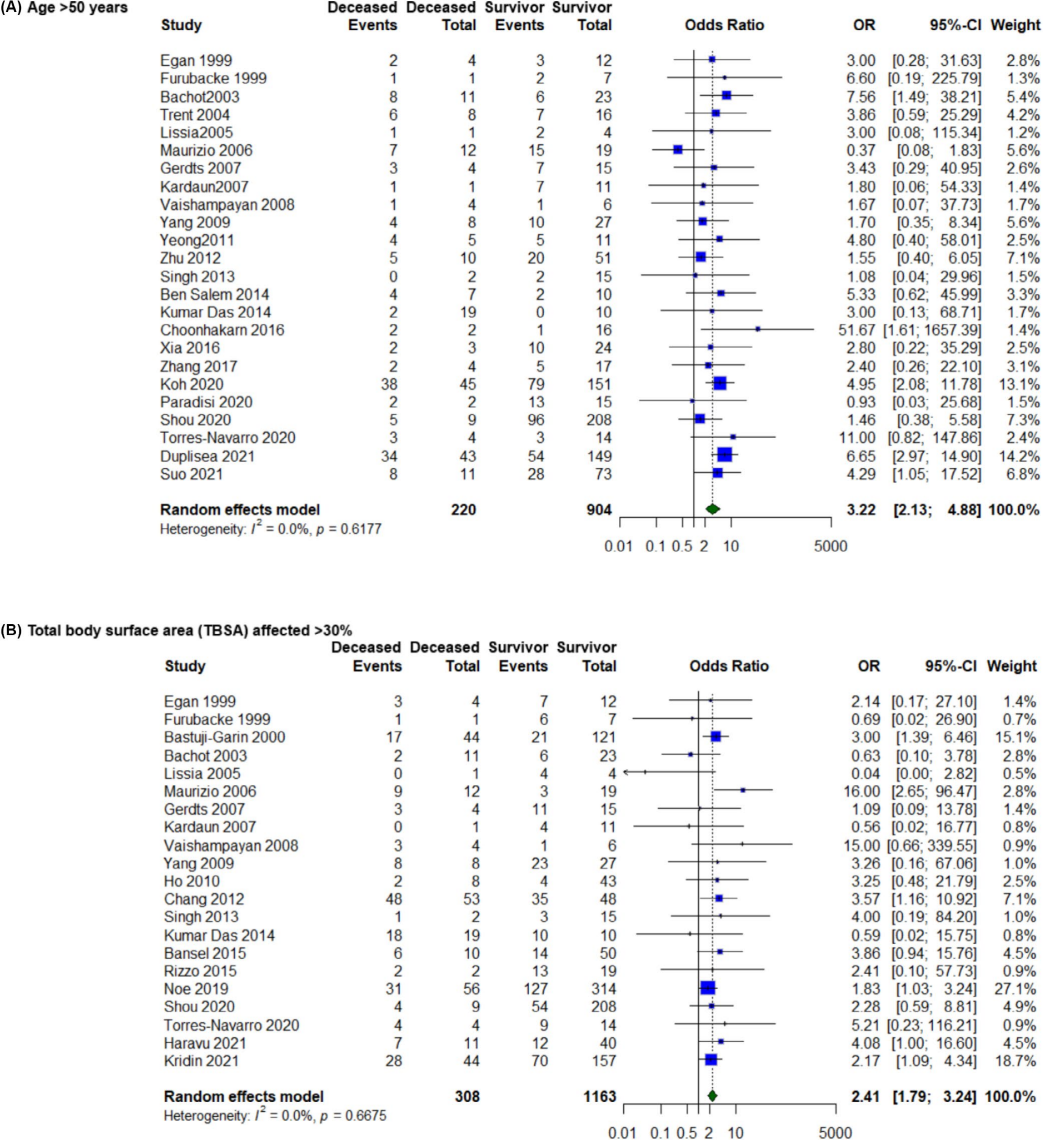
Forest plots illustrating the association between mortality in Stevens–Johnson syndrome/Toxic Epidermal Necrolysis patients and SCORTEN variables with alternate proposed cut‐offs. (A) Age > 50 years. (B) Total body surface area (TBSA) affected > 30%.

In total, 32 studies comprising 2124 patients investigated the standard age cut‐off of > 40, while 24 studies with 1124 patients analysed an age cut‐off of > 50. Both cut‐offs were significant and independent predictors of mortality, with the > 40 year cut off demonstrating a stronger association (OR = 3.62, 95% CI 2.71–4.84, *p* < 0.01) than the > 50 year cut off (OR = 3.22, 95% CI 2.13–4.88, *p* < 0.01) (Figure 3A).

19 studies including 1909 patients examined a TBSA cut‐off of > 10%, while 21 studies with 1471 patients analysed a TBSA cut‐off of > 30%. Once again, both cut‐offs were significant and independent predictors of mortality; however, the > 10% cut off demonstrated a stronger association (OR = 3.77, 95% CI 2.82–5.05, *p* < 0.01) compared to the > 30% cut off (OR = 2.41, 95% CI 1.79–3.24, *p* < 0.01) (Figure 3B).

### 3.4 Creatinine, White Cell Count, Eosinophil Count and Alanine Transaminase

**FIGURE 4:**
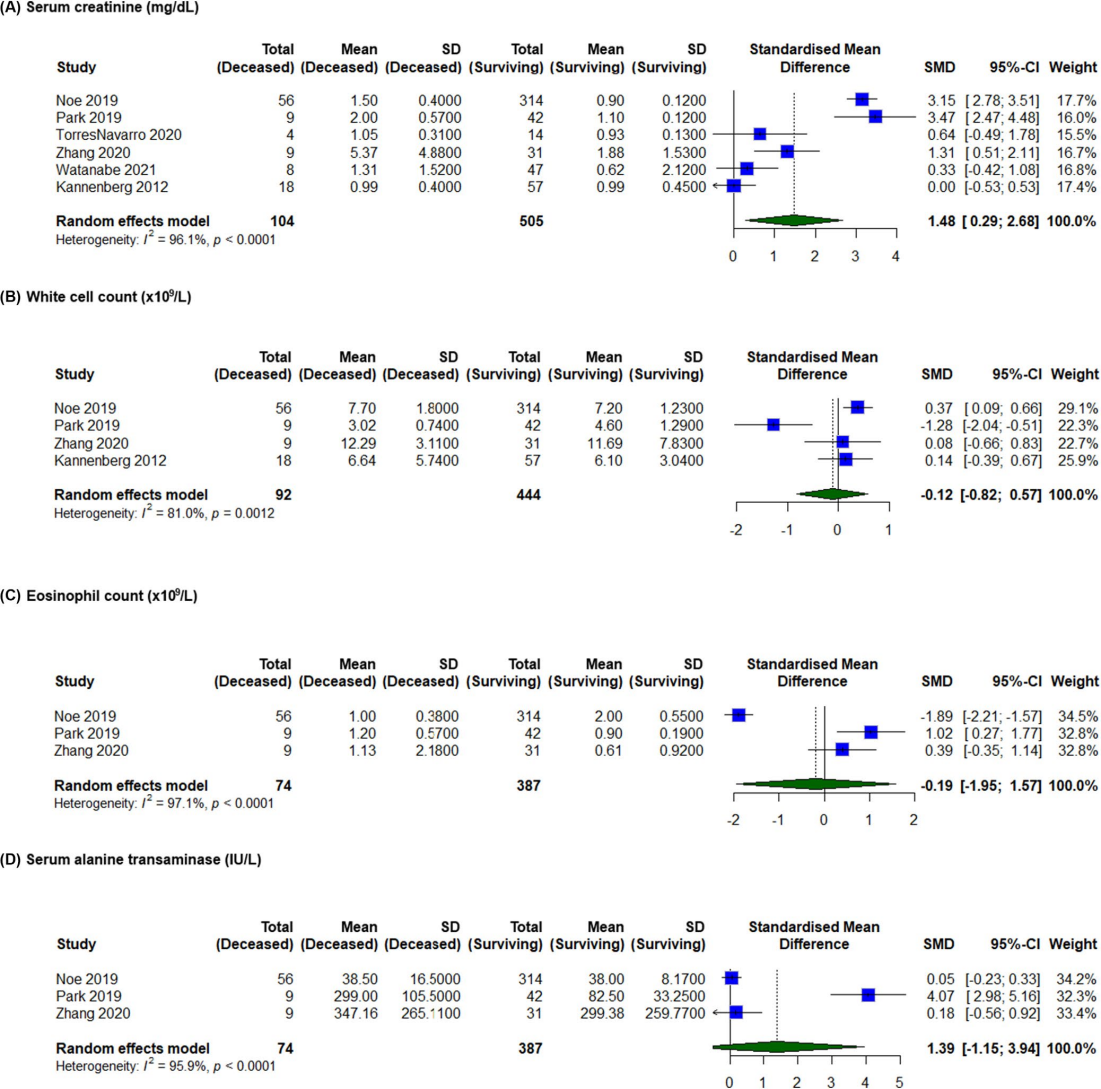
Forest plots illustrating the association between mortality in Stevens–Johnson syndrome/Toxic Epidermal Necrolysis patients and proposed novel prognostic markers. (A) Serum creatinine (mg/dL). (B) White cell count (×10^9^/L). (C) Eosinophil count (×10^9^/L). (D) Serum alanine transaminase (IU/L).

Four novel prognostic markers were reported consistently across enough studies to be meta‐analysable. A total of 6 studies including 609 patients compared serum creatinine levels between deceased and surviving patients. Elevated serum creatinine was associated with significantly increased risk of mortality (SMD = 1.47, 95% CI 0.29–2.65, *p* < 0.01) (Figure 4A).

A total of 4 studies including 536 patients compared serum white cell counts (WCC) between deceased and surviving patients. There was no significant association between WCC and risk of inpatient mortality (SMD = −0.12, 95% CI −0.82 to 0.58, *p* = 0.73) (Figure 4B).

A total of 3 studies including 461 patients compared serum eosinophil count between deceased and surviving patients. There was no significant association between eosinophil count and inpatient mortality (SMD = −0.19, 95% CI −1.95 to 1.58, *p* = 0.84). The same three studies also compared serum alanine transaminase (ALT) levels between deceased and surviving patients. Again, there was no significant association between ALT levels and inpatient mortality (SMD = 1.39, 95% CI −1.15 to 3.94, *p* = 0.28) (Figure 4C,D).

### 3.5 Albumin, Haemoglobin and Bilirubin

A 2020 Chinese study involving 40 SJS/TEN patients examined serum albumin levels between deceased (mean = 31.7 g/L, SD = 3.5) and surviving patients (mean = 32.6 g/L, SD = 4.5) and found no significant difference between the groups (*p* = 0.58) [26]. However, this study did find that deceased patients had significantly lower serum haemoglobin levels (deceased: mean = 101.0 g/L, SD = 29.2; survivors: mean = 120.8 g/L, SD = 22.0; *p* < 0.05) and significantly higher total serum bilirubin levels (deceased: mean = 60.6 mmol/L, SD = 60.0; survivors: mean = 21.1 mmol/L, SD = 19.1; *p* < 0.05) [26].

Due to a lack of studies reporting these markers as continuous variables, meta‐analysis could not be performed. Instead, some studies categorised these markers into dichotomous variables. For instance, the original SCORTEN cohort used a total serum bilirubin cutoff of 68.4 μmol/L but found no significant difference in mortality between patients above and below this threshold (*p* = 0.52) [6]. Similarly, a 2022 study from Thailand with 76 patients used a bilirubin cutoff of 68.4 μmol/L and did not find a significant association with mortality (*p* = 0.07) [27]. However, this study did identify significantly increased mortality risk in patients with haemoglobin levels < 100 g/L (RR = 3.93, 95% CI 1.31–11.85, *p* < 0.05) and those with albumin levels < 20 g/L (RR = 8.25, 95% CI 3.19–21.33, *p* < 0.001) [27]. These findings were corroborated by a 2021 South African study involving 75 patients, which reported a significantly higher mortality risk for patients with serum albumin < 25 g/L (OR = 8.50, 95% CI 2.43–29.75, *p* < 0.001) or haemoglobin < 100 g/L (OR = 4.68, 95% CI 1.52–14.47, *p* < 0.01) [28].

### 3.6 Risk of Bias

The QUIPS‐based risk of bias assessment highlighted consistent strengths across the domains of study participation, attrition, prognostic factor measurement, outcome measurement and statistical analysis (Table 1) [24]. Nearly all studies were rated as ‘low risk’ in these areas. However, 75.6% (34/45) studies were rated as ‘moderate risk’ with respect to assessment for confounding factors. This was due to inadequate adjustment for variables such as varying baseline characteristics between patients or differing treatment regimens.

**TABLE 1:** Risk of bias assessment for included studies using the Quality In Prognosis Studies (QUIPS) tool.

Study	Participation	Attrition	Predictors	Outcome	Confounders	Analysis
Bachot 2003	LOW	LOW	LOW	LOW	MODERATE	LOW
Bansal 2015	LOW	LOW	LOW	LOW	LOW	LOW
Bastuji‐Garin 2000	LOW	LOW	LOW	LOW	LOW	LOW
Ben Salem 2014	LOW	LOW	LOW	LOW	MODERATE	LOW
Chang 2012	LOW	LOW	LOW	LOW	MODERATE	LOW
Chantaphakul 2015	LOW	LOW	LOW	LOW	MODERATE	LOW
Choonhakarn 2016	LOW	LOW	LOW	LOW	MODERATE	LOW
Dorafshar 2007	LOW	LOW	LOW	LOW	LOW	LOW
Duplisea 2021	LOW	LOW	LOW	LOW	MODERATE	LOW
Egan 1999	LOW	LOW	LOW	LOW	MODERATE	LOW
Firoz 2012	LOW	LOW	LOW	LOW	LOW	LOW
Furubacke 1999	LOW	LOW	LOW	LOW	MODERATE	LOW
Gerdts 2007	LOW	LOW	LOW	LOW	MODERATE	LOW
Haravu 2021	LOW	LOW	LOW	LOW	MODERATE	LOW
Ho 2010	LOW	LOW	LOW	LOW	MODERATE	LOW
Kannenberg 2012	LOW	LOW	LOW	LOW	MODERATE	LOW
Kardaun 2007	LOW	LOW	LOW	LOW	MODERATE	LOW
Koh 2020	LOW	LOW	LOW	LOW	MODERATE	LOW
Kridin 2021	LOW	LOW	LOW	LOW	LOW	LOW
Kumar Das 2014	LOW	LOW	LOW	LOW	MODERATE	LOW
Lissia 2005	LOW	LOW	LOW	LOW	MODERATE	LOW
Noe 2019	LOW	LOW	LOW	LOW	MODERATE	LOW
Paradisi 2020	LOW	LOW	LOW	LOW	MODERATE	LOW
Park 2019	LOW	LOW	LOW	LOW	LOW	LOW
Rattanakaemakorn 2022	LOW	LOW	LOW	LOW	LOW	LOW
Rizzo 2015	LOW	LOW	LOW	LOW	MODERATE	LOW
Shou 2020	LOW	LOW	LOW	LOW	MODERATE	LOW
Singh 2013	LOW	LOW	LOW	LOW	MODERATE	LOW
Stella 2007	LOW	LOW	LOW	LOW	MODERATE	LOW
Suo 2021	LOW	LOW	LOW	LOW	MODERATE	LOW
Thakur 2021	LOW	LOW	LOW	LOW	MODERATE	LOW
Torres‐Navarro 2020	LOW	LOW	LOW	LOW	MODERATE	LOW
Trent 2004	LOW	LOW	LOW	LOW	MODERATE	LOW
Vaishampayan 2008	LOW	LOW	LOW	LOW	MODERATE	LOW
Wambier 2019	LOW	LOW	LOW	LOW	MODERATE	LOW
Wang 2016	LOW	LOW	LOW	LOW	MODERATE	LOW
Watanabe 2021	LOW	LOW	LOW	LOW	MODERATE	LOW
Wu 2015	LOW	LOW	LOW	LOW	LOW	LOW
Xia 2016	LOW	LOW	LOW	LOW	LOW	LOW
Yang 2009	LOW	LOW	LOW	LOW	MODERATE	LOW
Yeong 2011	LOW	LOW	LOW	LOW	LOW	LOW
Zavala 2017	LOW	LOW	LOW	LOW	MODERATE	LOW
Zhang 2017	LOW	LOW	LOW	LOW	MODERATE	LOW
Zhang 2020	LOW	LOW	LOW	LOW	LOW	LOW
Zhu 2012	LOW	LOW	LOW	LOW	MODERATE	LOW

*Note:* Risk of bias was assessed across six domains: study participation, attrition, predictor measurement, outcome measurement, consideration of confounding factors and statistical analysis/reporting. Each study was rated as having low, moderate or high risk of bias for each domain.

## 4 Discussion

The SCORTEN tool, developed in 2000, marked a significant advancement in the prognostic assessment of SJS/TEN [6]. It provided a systematic approach to evaluating disease severity by incorporating seven clinical variables. SCORTEN's simplicity contributed to its widespread adoption in clinical practice, and over two decades, numerous studies have validated its efficacy in mortality prediction and clinical decision‐making [29, 30]. However, despite its widespread use, concerns have been raised regarding its accuracy and relevance in current clinical settings [15, 31].

Contemporary criticisms of SCORTEN are threefold. Firstly, the score's original development and validation were based on an old patient cohort treated between 1979 and 1998 [6]. Since then, management paradigms in SJS/TEN have continued to evolve, including changes in treatment strategies, advancements in supportive care capabilities, and the introduction of modern therapies including intravenous immunoglobulin (IVIG) and TNF‐α inhibitors such as etanercept [32, 33]. As survival outcomes continue to improve, the applicability of SCORTEN to contemporary patient populations warrants reassessment [21, 34]. Secondly, SCORTEN consists of parameters that may be subject to variability and subjectivity in measurement. For example, heart rate can be influenced by factors such as pain, anxiety and medications, leading to fluctuations that may not accurately reflect clinical status. Similarly, estimating TBSA involvement can be challenging and subjective, particularly in cases where there is partial or evolving epidermal detachment [35]. This variability may introduce inconsistencies in the application of SCORTEN and affect its predictive accuracy. Thirdly, in the construction of SCORTEN, continuous variables such as age and laboratory parameters were summarised and reduced into dichotomous variables. While this simplification may enhance ease of use, it also results in a loss of information. Dichotomizing continuous variables can lead to the masking of subtle differences in risk, whilst exaggerating incremental variable changes for patients who fall in the borderline region [6, 13].

Despite these criticisms, our meta‐analysis found that all SCORTEN parameters remained valid and independent predictors of mortality. Among them, serum BUN exhibited the strongest association (OR = 6.52, 95% CI 4.80–8.86), followed by bicarbonate levels (OR = 5.78, 95% CI 3.77–8.85).

More novel prognostic tools have been devised since the implementation of SCORTEN. Aimed at streamlining prognostication and reducing reliance on biochemical markers, the ABCD‐10 score was developed and validated in a modern multicenter cohort of 370 North American patients [7]. It used an alternative cut‐off age of 50, however our analysis revealed that the conventional SCORTEN cutoff actually demonstrated a stronger association with mortality (OR 3.62 vs. 3.22) [7]. Similarly, the Auxiliary score, derived from an Australian burn‐center cohort of 27 patients, utilised an alternative TBSA cutoff of 30% [8]. Yet again, the traditional SCORTEN cutoff of 10% proved superior (OR 3.77 vs. 2.41), possibly due to a higher sample size of studies which assessed the > 10% TBSA threshold [8]. This underscores the importance of capturing variations in TBSA involvement with greater granularity, as even small differences in cutoff points can impact the predictive accuracy of prognostic models. Furthermore, the Clinical Risk Score for TEN (CRISTEN) was developed specifically to aid in rapid prognostication prior to receiving biochemical results [36]. Utilising an age cut‐off of 65, this was reported to have a stronger association with mortality compared to SCORTEN (OR 3.95 vs. 3.62) [36]. However, CRISTEN has been validated less extensively and demonstrated a slightly lower predictive accuracy than SCORTEN overall [36].

Conversely, efforts have been made to refine and extend SCORTEN itself in order to strengthen its prognostic value. Re‐SCORTEN, devised in 2021, includes the ratio of red cell distribution width to haemoglobin (RDW/Hb) as a further prognostic marker (OR 3.55) in addition to the existing SCORTEN criteria [16]. Re‐SCORTEN has been found to have significantly superior predictive accuracy for in‐hospital mortality than SCORTEN alone, though it too has been validated less widely [16]. This highlights an inherent tension between ease of use and predictive accuracy in these prognostic tools.

Considering the complex pathophysiology and varied clinical trajectory of SJS/TEN, there is a growing interest in identifying novel prognostic markers to enhance risk stratification and improve patient outcomes [16, 36]. Recently, renal failure has been increasingly recognised as a pertinent predictor of mortality. Mechanisms underpinning acute renal failure in SJS/TEN are multifaceted. Firstly, it is postulated that nephrotoxic cytokines released during SJS/TEN directly induce kidney injury [17, 22, 37]. Moreover, epidermal detachment can lead to significant fluid losses, contributing to renal hypoperfusion and subsequent injury [38]. While impaired renal function is indirectly reflected in SCORTEN parameters including serum BUN and serum bicarbonate, the direct relationship remains contentious. The ABCD‐10 model incorporated prior dialysis status as part of its prognostic model, assigning it the greatest weight out of all parameters [7]. However, subsequent studies did not demonstrate ABCD‐10 to be superior to SCORTEN [11]. Our findings support the role of serum creatinine as a surrogate indicator of acute renal failure, where its incorporation into predictive models may offer additive benefits for prognostic precision (SMD = 1.47, 95% CI 0.29–2.65, *p* < 0.01) [17, 35].

In addition to creatinine, WCC has emerged as another potential prognostic marker, acting as a surrogate for systemic infection and sepsis, which remains the predominant driver of SJS/TEN mortality [39]. However, due to its nonspecific nature and rapid fluctuations, WCC often proves unreliable [40, 41]. Indeed, we did not find a significant association between WCC and mortality in our meta‐analysis (SMD = −0.12, 95% CI −0.82 to 0.58, *p* = 0.73). Another haematological marker, eosinophil, is often elevated in Drug Reaction with Eosinophilia and Systemic Symptoms (DRESS). Whilst SJS/TEN and DRESS are two separate entities with distinct clinicopathological hallmarks, there have been isolated reports of overlapping syndromes [42]. It is possible that such a process would increase the risk of mortality from both conditions; however, our meta‐analysis failed to find any significant correlation between eosinophil count in SJS/TEN patients and risk of mortality (SMD = −0.19, 95% CI −1.95 to 1.58, *p* = 0.84). While sporadic mentions of other novel prognostic markers exist for SJS/TEN, the scarcity of comparable data and heterogeneity of patient populations and treatments precludes meta‐analysis [43, 44]. This underscores the ongoing challenges in identifying and integrating novel prognostic markers into comprehensive prognostic models for SJS/TEN.

## 5 Limitations and Future Directions

Our review is subject to several limitations. Firstly, the quality of the studies varied, as illustrated by many studies demonstrating inadequate control for confounding variables based on the QUIPS tool. The majority of included studies were retrospective, introducing limitations such as selection bias and incomplete data reporting (Table S1) [45]. Additionally, there was no standardised timeline for the assessment of SCORTEN variables across studies. While these variables should ideally be collected upon initial presentation, various confounding clinical factors, such as timing of presentation and diagnosis, may influence their assessment [29]. Moreover, certain SCORTEN parameters, such as heart rate and TBSA involvement, are prone to subjectivity in their evaluation [46]. Another major confounder is the difference in treatment protocol across studies. There are no standardised therapies for SJS/TEN, and SCORTEN has been widely adopted as a prognostic benchmark in therapeutic studies, potentially leading to varying mortality rates due to heterogeneous treatment strategies [14]. We made the decision to analyse all patient data regardless of treatment approach, as this reflects real‐world practice where SCORTEN is typically calculated on presentation prior to treatment initiation.

Important directions for future research include standardising the measurement schedules for parameters in prognostic models. We hypothesize that current variability in SCORTEN measures arises from differences in the timing and method of data collection; thus, standardising this process may further enhance the model's stability [11, 31]. This may entail the integration of novel markers, deprecating more variable parameters and transitioning to continuous input variables. While challenging due to the rarity of SJS/TEN, prospective studies with large sample sizes and standardised data collection protocols would provide a more robust evidence base for prognostic models [13]. Furthermore, as survival rates in SJS/TEN continue to improve with the refinement of supportive care capabilities, there has been a paradigm shift towards evaluating more nuanced morbidity outcomes such as hospital length‐of‐stay, pain scores and time to re‐epithelialization [47, 48, 49]. This broader spectrum of clinical outcomes will allow future research to provide a more comprehensive evaluation of disease severity and treatment efficacy in the contemporary burn center era. Addressing these considerations for future research will allow researchers to continue to advance our knowledge of SJS/TEN, improve risk stratification and treatment decision‐making, and ultimately enhance the care and outcomes of patients affected by this rare and severe condition.

## 6 Conclusion

In conclusion, our systematic review confirms that all seven SCORTEN variables remain robust, independent predictors of mortality in SJS/TEN patients, reinforcing its continued utility as a bedside prognostic tool. Elevated serum creatinine is also a promising adjunct marker that may enhance risk stratification, although white cell count, eosinophil count, and transaminases did not demonstrate consistent prognostic value. Moving forward, prospective studies with standardised measurement protocols and exploration of additional continuous or composite markers will be critical to refine prognostic models, optimise therapeutic decisions and improve outcomes.

## Funding

The authors have nothing to report.

## Conflicts of Interest

The authors declare no conflicts of interest.

## Supporting information


**Appendix S1:** ajd70082‐sup‐0001‐AppendixS1.pdf.

## Data Availability

The data that support the findings of this study are available from the corresponding author upon reasonable request.
