# Supplementary material for: SCORTEN and Novel Prognostic Markers in Stevens–Johnson Syndrome/Toxic Epidermal Necrolysis: A Systematic Review and Meta‐Analysis
Source: Australas J Dermatol. 2026 Mar 6;67(3):e143–53. doi: 10.1111/ajd.70082 (PMC13176803; doi:10.1111/ajd.70082)
Supplement: Supplementary file 1 — Appendix S1: ajd70082‐sup‐0001‐AppendixS1.pdf. [file AJD-67-e143-s001.pdf]

Supplementary Material

Supplementary Figure 1

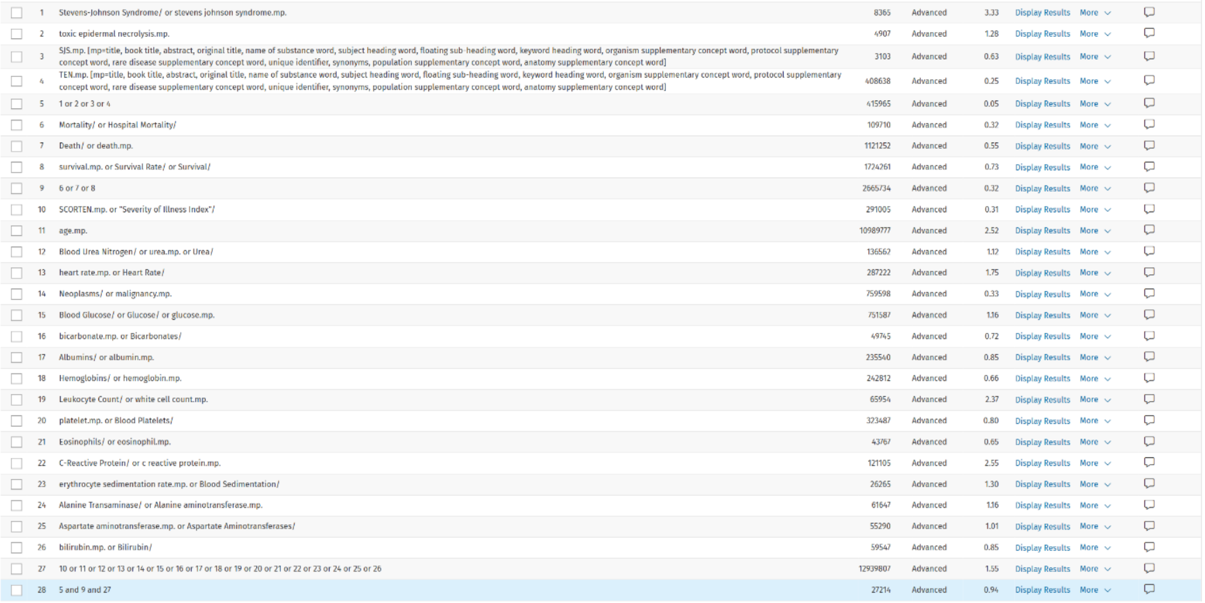

1	Stevens-Johnson Syndrome/ or stevens johnson syndrome.mp.	8365	Advanced	3.33	Display Results	More	
2	toxic epidermal necrolysis.mp.	4907	Advanced	1.28	Display Results	More	
3	SJS.mp. [mp=title, book title, abstract, original title, name of substance word, subject heading word, floating sub-heading word, keyword heading word, organism supplementary concept word, protocol supplementary concept word, rare disease supplementary concept word, unique identifier, synonyms, population supplementary concept word, anatomy supplementary concept word]	3103	Advanced	0.63	Display Results	More	
4	TEN.mp. [mp=title, book title, abstract, original title, name of substance word, subject heading word, floating sub-heading word, keyword heading word, organism supplementary concept word, protocol supplementary concept word, rare disease supplementary concept word, unique identifier, synonyms, population supplementary concept word, anatomy supplementary concept word]	408638	Advanced	0.25	Display Results	More	
5	1 or 2 or 3 or 4	415965	Advanced	0.05	Display Results	More	
6	Mortality/ or Hospital Mortality/	109710	Advanced	0.32	Display Results	More	
7	Death/ or death.mp.	1121252	Advanced	0.55	Display Results	More	
8	survival.mp. or Survival Rate/ or Survival/	1724261	Advanced	0.73	Display Results	More	
9	6 or 7 or 8	2665734	Advanced	0.32	Display Results	More	
10	SCORTEN.mp. or "Severity of Illness Index"/	291005	Advanced	0.31	Display Results	More	
11	age.mp.	10989777	Advanced	2.52	Display Results	More	
12	Blood Urea Nitrogen/ or urea.mp. or Urea/	136562	Advanced	1.12	Display Results	More	
13	heart rate.mp. or Heart Rate/	287222	Advanced	1.75	Display Results	More	
14	Neoplasms/ or malignancy.mp.	759598	Advanced	0.33	Display Results	More	
15	Blood Glucose/ or Glucose/ or glucose.mp.	751587	Advanced	1.16	Display Results	More	
16	bicarbonate.mp. or Bicarbonates/	49745	Advanced	0.72	Display Results	More	
17	Albumins/ or albumin.mp.	235540	Advanced	0.85	Display Results	More	
18	Hemoglobins/ or hemoglobin.mp.	242812	Advanced	0.66	Display Results	More	
19	Leukocyte Count/ or white cell count.mp.	65954	Advanced	2.37	Display Results	More	
20	platelet.mp. or Blood Platelets/	323487	Advanced	0.80	Display Results	More	
21	Eosinophils/ or eosinophil.mp.	43767	Advanced	0.65	Display Results	More	
22	C-Reactive Protein/ or c reactive protein.mp.	121105	Advanced	2.55	Display Results	More	
23	erythrocyte sedimentation rate.mp. or Blood Sedimentation/	26265	Advanced	1.30	Display Results	More	
24	Alanine Transaminase/ or Alanine aminotransferase.mp.	61647	Advanced	1.16	Display Results	More	
25	Aspartate aminotransferase.mp. or Aspartate Aminotransferases/	55290	Advanced	1.01	Display Results	More	
26	bilirubin.mp. or Bilirubin/	59547	Advanced	0.85	Display Results	More	
27	10 or 11 or 12 or 13 or 14 or 15 or 16 or 17 or 18 or 19 or 20 or 21 or 22 or 23 or 24 or 25 or 26	12939807	Advanced	1.55	Display Results	More	
28	5 and 9 and 27	27214	Advanced	0.94	Display Results	More	

Supplementary Figure 2

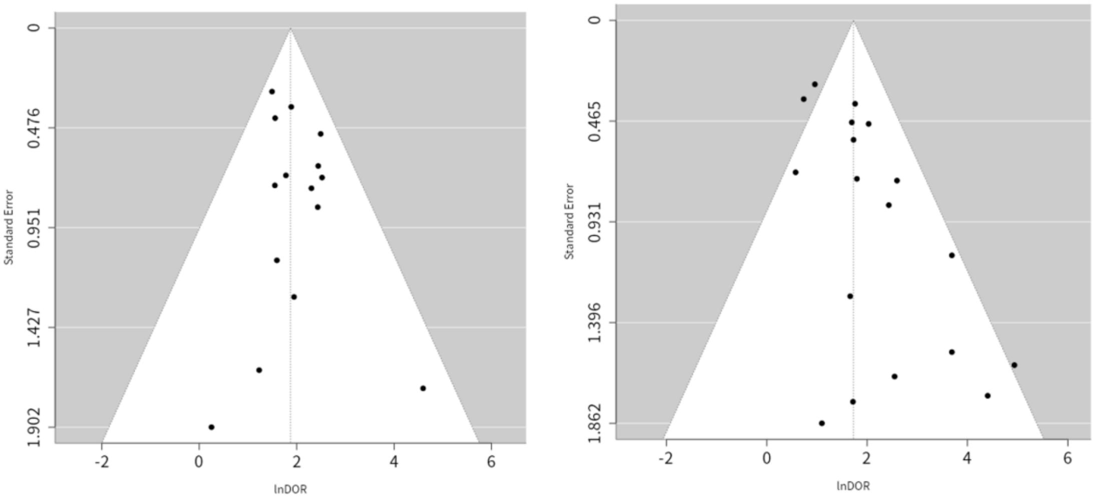

A: Funnel plot: Serum Urea >10mmol/L B: Funnel plot: Serum Bicarbonate <20mmol/L

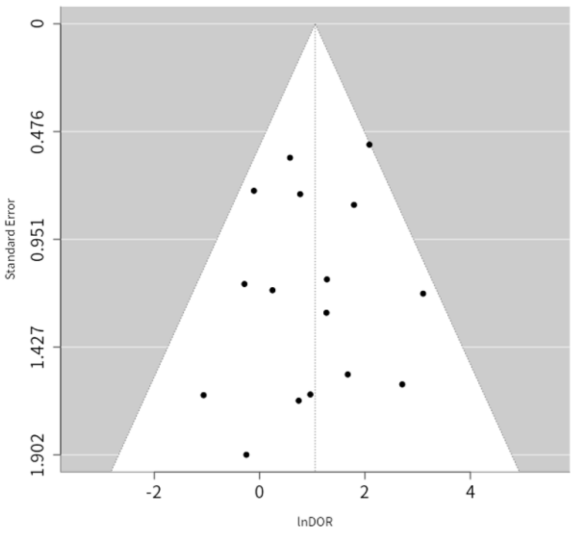

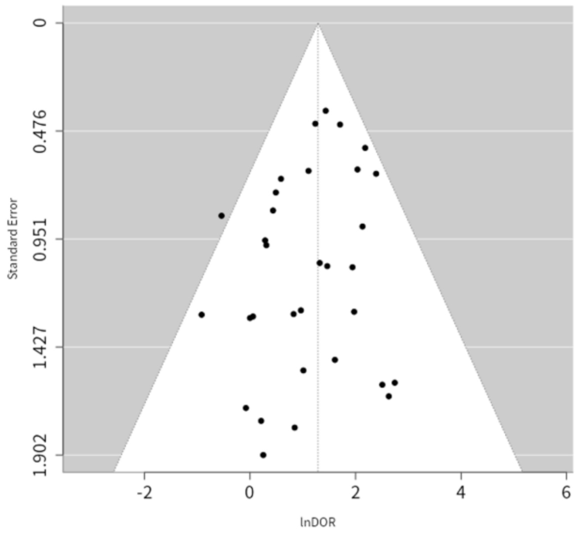

C: Funnel plot: Serum Glucose >14mmol/L

D: Funnel plot: Age >40 years

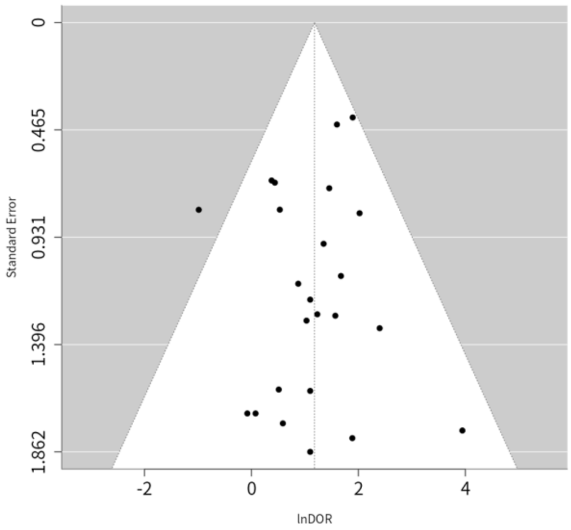

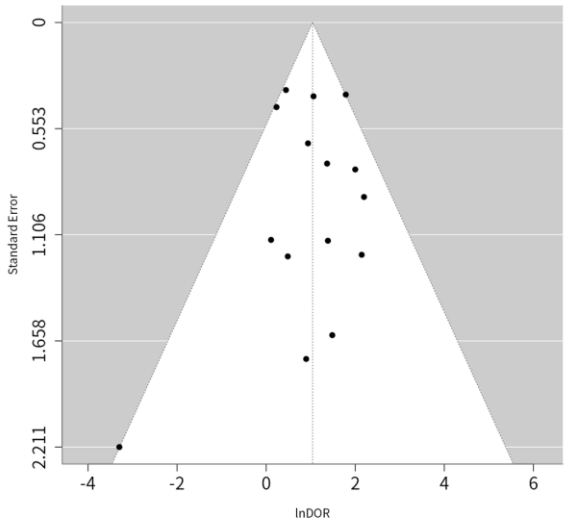

E: Funnel plot: Age >50 years

F: Funnel plot: Heart rate >120 beats per minute

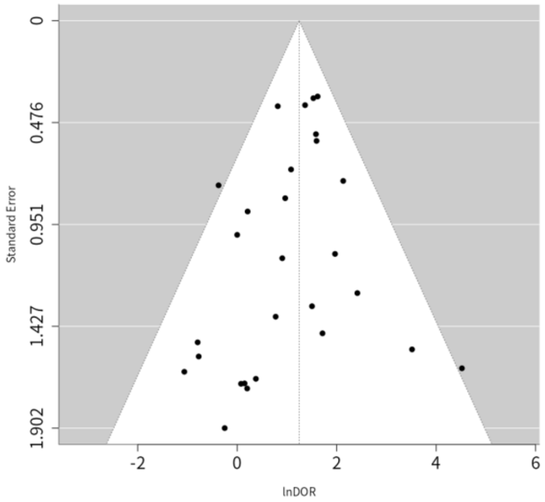

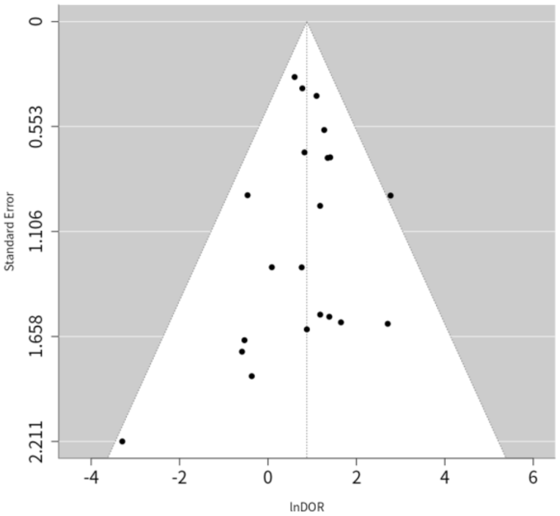

G: Funnel plot: Presence of active malignancy

H: Funnel plot: Total body surface area >30%

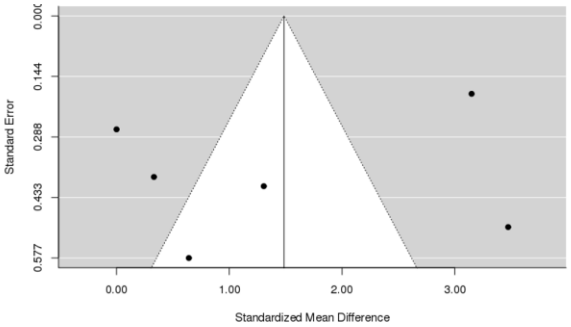

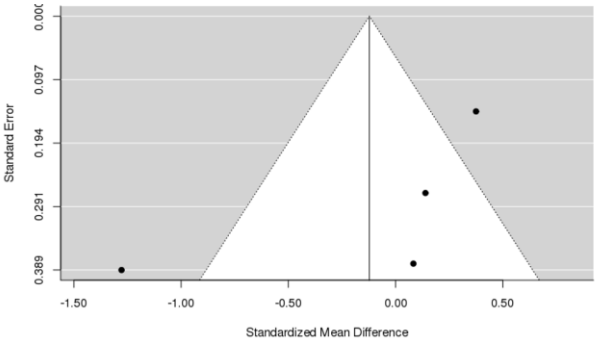

I: Funnel plot: Serum Creatinine Level

J: Funnel plot: Serum White Cell Count

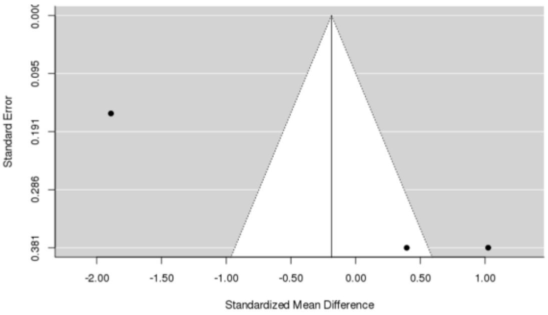

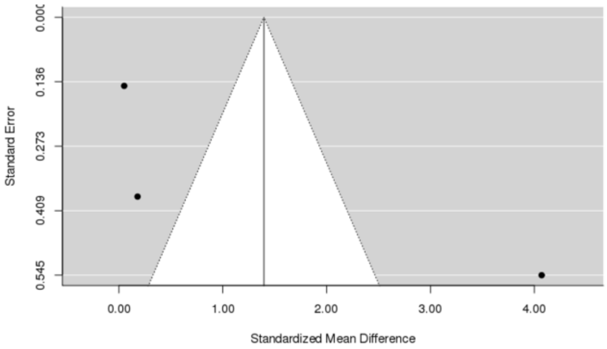

K: Funnel plot: Serum Eosinophil Count

L: Funnel plot: Serum Alanine Transaminase

Supplementary Table 1

Author	Year	Country	Design	Total	Deceased (n, %)	Females (n, %)	Age (mean, SD)
Bachot	2003	France	PCS	34	11 (32.4%)	22 (64.7%)	47 (21)
Bansal	2015	India	PCS	60	10 (16.7%)	NR	23.3 (4.3)
Bastuji-Garin	2000	France	RCS	165	44 (26.7%)	78 (47.3%)	42.3 (19.8)
Ben Salem	2014	Tunisia	RCS	17	7 (41.2%)	14 (82.4%)	42.9 (19.8)
Chang	2012	Taiwan	RCS	101	53 (52.5%)	57 (56.4%)	56.1 (23.1)
Chantaphakul	2015	Thailand	RCS	43	3 (7.0%)	18 (41.9%)	49.5
Choonhakarn	2016	Thailand	RCS	18	2 (11.1%)	9 (50.0%)	49.7 (20.6)
Dorafshar	2007	United States	RCS	48	13 (27.1%)	29 (60.4%)	46.7 (27.3)
Duplisea	2021	United States	RCS	192	43 (22.4%)	117 (60.9%)	46
Egan	1999	United States	RCS	16	4 (25.0%)	11 (68.8%)	42.4 (21.6)
Firoz	2012	United States	PCS	82	24 (29.3%)	38 (46.3%)	45.1 (20.4)
Furubacke	1999	Sweden	RCS	8	1 (12.5%)	5 (62.5%)	45
Gerdts	2007	Netherlands	RCS	19	4 (21.1%)	11 (57.9%)	45.6 (23.3)
Haravu	2021	United States	RCS	51	11 (21.6%)	28 (54.9%)	47.2 (24.4)
Ho	2010	Taiwan	RCS	51	8 (15.7%)	22 (43.1%)	62.7 (18.9)
Kannenberg	2012	South Africa	PCS	75	24 (32.0%)	59 (78.7%)	39.9 (10.6)
Kardaun	2007	Netherlands	RCS	12	1 (8.3%)	6 (50.0%)	55.6 (21.8)
Koh	2020	Singapore	RCS	196	45 (23.0%)	116 (59.2%)	56
Kridin	2021	Europe	RCS	201	44 (21.9%)	134 (66.7%)	51.0 (19.3)
Kumar Das	2014	Bangladesh	PCS	29	19 (65.5%)	9 (31%)	26.8 (12.7)
Lissia	2005	Italy	PCS	5	1 (20.0%)	1 (20.0%)	65.8 (18.9)
Stella	2007	Italy	RCS	31	12 (38.7%)	23 (74.2%)	58.8 (16.9)
Noe	2019	United States	RCS	370	56 (15.1%)	195 (52.7%)	49.0 (19.1)
Paradisi	2020	Italy	PCS	17	2 (11.8%)	8 (47.1%)	66.4 (19.4)
Park	2019	South Korea	RCS	51	9 (17.6%)	23 (45.1%)	71
Rattanakaemakorn	2022	Thailand	RCS	76	10 (13.2%)	40 (52.6%)	52 (18.1)
Rizzo	2015	United States	RCS	21	2 (9.5%)	10 (47.6%)	10.9 (4.8)
Shou	2020	China	RCS	217	9 (4.1%)	99 (45.6%)	48.2 (1.3)
Singh	2013	India	RCS	17	2 (11.8%)	5 (29.4%)	32.1 (16.2)
Suo	2021	China	RCS	84	11 (13.1%)	46 (54.8%)	49.2 (16.1)
Thakur	2021	India	RCS	51	11 (21.6%)	24 (47.1%)	38.2 (17.6)
Torres-Navarro	2020	Spain	RCS	18	4 (22.2%)	15 (83.3%)	43.7 (25.7)
Trent	2004	United States	RCS	24	8 (33.3%)	8 (33.3%)	52.7 (17.7)
Vaishampayan	2008	India	PCS	10	4 (40.0%)	6 (60.0%)	32.1 (22.8)
Wambier	2019	Brazil	RCS	69	12 (17.4%)	22 (31.9%)	32
Wang	2016	China	RCS	88	6 (6.8%)	48 (54.5%)	45 (18)
Watanabe	2021	Japan	RCS	55	8 (14.5%)	28 (50.9%)	61
Wu	2015	Taiwan	RCS	517	55 (10.6%)	273 (52.8%)	49.9 (21.7)
Xia	2016	China	RCS	27	3 (11.1%)	15 (55.6%)	43.6 (19.3)
Yang	2009	China	RCS	35	8 (22.9%)	14 (40.0%)	43.7 (22.8)
Yeong	2011	Taiwan	RCS	16	5 (31.3%)	8 (50.0%)	58 (23)
Zavala	2017	United States	RCS	128	26 (20.3%)	76 (59.4%)	44.5 (23.8)
Zhang	2017	China	RCS	21	4 (19.0%)	11 (52.4%)	33
Zhang	2020	China	RCS	40	9 (22.5%)	15 (37.5%)	49.4 (21.2)
Zhu	2012	China	RCS	61	10 (16.4%)	26 (42.6%)	46.4 (19.0)

Figure Captions:

Supplementary Fig 1

Ovid MEDLINE search strategy. Searches were conducted from database inception to 31 March 2024 and limited to human studies published in English. The full search strategy is provided in Appendix S1.

Supplementary Fig 2

Funnel plots assessing risk of small-study effects for (A) Serum urea >10mmol/L (B) Serum bicarbonate <20mmol/L (C) Serum glucose >14mmol/L (D) age >40 years (E) Age >50 years (F) Heart rate >120 beats per minute (G) Malignancy status (H) Total body surface area affected >30% (I) Serum creatinine (mg/dL) (J) White cell count (x109/L) (K) Eosinophil count (x109/L) (L) Serum alanine transaminase (IU/L)

Table Caption:

Supplementary Table 1

Characteristics of included studies (n = 45). Country, study design, cohort size, mortality, sex distribution, mean age, and study quality are summarised for all studies included in the systematic review and meta-analysis. Age is reported as mean (SD) where available; where SD was not reported, mean age only is shown. PCS = prospective cohort study; RCS = retrospective cohort study; SD = standard deviation; NR = not reported.

Appendix S1. Search strategy

Ovid MEDLINE (inception to 31 March 2024)

(("Stevens-Johnson Syndrome" OR "toxic epidermal necrolysis" OR SJS OR TEN)

AND

("mortality" OR "hospital mortality" OR death OR survival OR "survival rate")

AND

(SCORTEN OR "severity of illness index" OR age OR "blood urea nitrogen" OR urea OR "heart rate" OR malignancy OR neoplasms OR glucose OR "blood glucose" OR bicarbonate OR albumin OR hemoglobin OR "white cell count" OR "leukocyte count" OR platelet OR "blood platelets" OR eosinophil OR "c-reactive protein" OR "erythrocyte

sedimentation rate" OR "alanine transaminase" OR "aspartate aminotransferase" OR bilirubin))

Limits applied: Humans; English language.

Timespan: All years.

Equivalent search strategies were adapted for Embase and the Cochrane Library using database-specific subject headings and keywords
